# Supplementary material for: Using partial least squares to identify a dietary pattern associated with obesity in a nationally-representative sample of Canadian adults: Results from the Canadian Community Health Survey—Nutrition 2015
Source: PLoS One. 2021 Aug 5;16(8):e0255415. doi: 10.1371/journal.pone.0255415 (PMC8341606; doi:10.1371/journal.pone.0255415)
Supplement: S1 Table — (PDF) [file pone.0255415.s001.pdf]

S1 Table. Scoring criteria for the Dietary Guidelines for Americans Adherence Index (DGAI) for individuals with 2000 kcal/day estimated energy requirement (EER)

Components					
	Scoring Criteria			Scoring Criteria	
	0 Point	1.0 point		0 Point	1.0 point
**Food Intake Sub-score**			**Healthy Choice Sub-score**		
Dark green vegetable (cups[1]/week)	0	≥ 1.5	Whole grain (% of grains)	0	≥ 50%
Red/orange vegetables (cup/week)	0	≥ 5.5	Dietary fiber density (gram/1000kcal)	0	≥ 14
Legumes (cup/week)	0	≥ 1.5	Total fat (% Energy)	≤ 10%, ≥ 45%	≥ 20%, ≤ 35%
Starchy vegetables (cup/week)[2]	0	5.0	Saturated fatty acid (% Energy)	≥ 15%	≤ 10%
Other vegetables (cup/week)	0	≥ 4.0	Cholesterol intake (mg/day)	≥ 450	≤ 300
Fruits (cup/day)	0	≥ 2	Low-fat dairy, and meat products (%)	0%	≥ 75%
Variety of fruits and vegetables (number of components)	0	6.0	Sodium (mg/day)	≥ 3450	≤ 2300
Grains (oz equivalent[1]/day)[2]	0	6.0	Alcohol (drinks/day)[3]	≥ 1.5	≤ 1.0
Meat and beans (oz equivalent/day)[2]	0	26			
Dairy (cup/day)[2]	0	3			
Added sugar (% Energy)	≥ 9%	≤ 6.0%			

[1]One cup is defined as 237 ml (US), 0.946 cup in metric unit; 1 oz=28.35 grams
[2]An overconsumption penalty was imposed by reducing the score proportional to the amount of overconsumption up to 1.25 times higher than the recommended intake. Intakes ≥1.25 times the recommended amount were scored as 0.5.
[3]One drink =118 ml wine; 355 ml beer; or 45 ml distilled spirit
