# Supplementary material for: Using partial least squares to identify a dietary pattern associated with obesity in a nationally-representative sample of Canadian adults: Results from the Canadian Community Health Survey—Nutrition 2015
Source: PLoS One. 2021 Aug 5;16(8):e0255415. doi: 10.1371/journal.pone.0255415 (PMC8341606; doi:10.1371/journal.pone.0255415)
Supplement: S2 Table — (PDF) [file pone.0255415.s002.pdf]

S2 Table. Percent variation explained from the wPLS-derived dietary pattern for two scenarios: energy density, total fat intake and fiber density as response variables (Model A); and energy density, saturated fat intake and fiber density as response variables (Model B).

wPLS Model	Percent of Variation Explained in Response	Percent of Variation Explained in Predictors
Model A (energy density, total fat intake and fiber density)	49.50%	10.40%
Model B (energy density, saturated fat intake and fiber density)	46.40%	10.29%
