# Supplementary material for: Using partial least squares to identify a dietary pattern associated with obesity in a nationally-representative sample of Canadian adults: Results from the Canadian Community Health Survey—Nutrition 2015
Source: PLoS One. 2021 Aug 5;16(8):e0255415. doi: 10.1371/journal.pone.0255415 (PMC8341606; doi:10.1371/journal.pone.0255415)
Supplement: S3 Table — (PDF) [file pone.0255415.s003.pdf]

S3 Table. Predictor variables used in weighted partial least squares (PLS) analysis in the Canadian Community Health Survey-Nutrition 2015.

Predictor food groups	Corresponding BNS Food Code(s)
1. Fast food	38B, 170A, 170B, 170C, 170D, 214A, 214B, 219A, 219B, 219C, 219D, 219E, 219F
2. Carbonated drinks (including energy drinks, sports drinks and vitamin water)	46A, 46B, 46C, 46E, 46F, 46G
3. Salty snacks	38A, 42B
4. Baked goods (including granola bars)	4C, 4E, 4F, 7A, 7B, 7C, 8A, 8B, 8C, 140D, 140F, 150A, 150B, 150C, 150D150E, 150F, 150G, 150H, 150I
5. Alcohol	47B, 48A, 49A, 49B
6. Solid fats	13A, 13B, 13C, 13D, 17A, 18A, 18B, 20A, 21B, 21C
7. Sweets	9A, 9B, 9C, 43A, 43B, 43C, 44A, 46D, 160B, 205A,
8. Coffee	51B
9. Sugar	41A, 41B, 41C, 41D
10. Full-fat milk products	10A
11. Sauce	50C, 50D, 50E
12. Processed meat	25D, 25E, 30A, 32A
13. Cheese	14A, 14B, 14C, 14D
14. Nuts and seeds	33A, 33B, 33C
15. Refined breads and cereals	2A, 4A, 4B, 6A
16. Milk substitutes (goat milk, sheep milk, buttermilk, etc.)	10E, 10F, 10G, 10H, 10I, 10K
17. Pancakes and waffles	4D, 140E
18. Veal, lamb, pork	23A, 23B, 24A, 24B, 25A, 25B, 25C
19. Low-fat milk products	10B, 10C, 10D
20. Seasoning	50F, 53A, 53B
21. Eggs	16A, 16B
22. Fruit juice	45A
23. Beef and organ meats	22A, 22B, 22C, 28A, 28B, 29A, 31A
24. Poultry	27A, 28B, 28C, 28D, 28E, 28F
25. Starchy vegetables	36G, 36K, 39A
26. Vegetable oils	21A
27. Energy bars, energy drinks and meal replacements	54A, 54B, 54C
28. Seafood and shellfish	34A, 34B, 35A
29. Tea	51A
30. Water	51C
31. Plant-based milks	10J
32. Yogurt	15A, 15B
33. Mixed dishes	50A, 50B, 99A, 215A, 216A
34. Pasta and rice	1A, 1B, 1C
35. Legumes and soy	37A, 37B
36. Dark green vegetables	36B, 36H
37. Whole grain breads and cereals	3A, 3B, 5A
38. "Other" vegetables (including vegetable juice)	36A, 36C, 36F, 36I, 36J 36L, 36P, 360
39. Orange and red vegetables	36E, 36M, 36N
40. Whole fruit	40A, 40B, 40C, 40D, 40E, 40F, 40G, 40H, 40I, 40J, 40K, 40L

Note: additions to the Bureau of Nutritional Sciences (BNS) Food Codes in the 2015 Canadian Community Health Survey-Nutrition include: 46E, 46F, 46G, 7C, 54A, 54B, 54C[161]. Milk substitutes were also re-categorized in 2015 into plant-based milks (10J) and other dairy milk substitutes (such as buttermilk, sheep milk, etc. in 10E, 10F, 10G, 10H, 10I, 10K).
