# Supplementary material for: Using partial least squares to identify a dietary pattern associated with obesity in a nationally-representative sample of Canadian adults: Results from the Canadian Community Health Survey—Nutrition 2015
Source: PLoS One. 2021 Aug 5;16(8):e0255415. doi: 10.1371/journal.pone.0255415 (PMC8341606; doi:10.1371/journal.pone.0255415)
Supplement: S4 Table — Results are across quartiles of the energy-dense, high-fat and low-fiber density (“obesogenic”) dietary pattern score from the wPLS (weighted partial least squares) model and B). (PDF) [file pone.0255415.s004.pdf]

S4 Table. Odds ratios and 95% confidence intervals for the likelihood of “healthy” or “unhealthy” obesity, with normal weight (BMI<30) as reference. Results are across quartiles of the energy-dense, high-fat and low-fiber density (“obesogenic”) dietary pattern score from the wPLS (weighted partial least squares) model and B).

	Q1 (Ref; Healthiest)	Q2	Q3	Q4 (Least Healthy)	P-trend
Normal weight (BMI<30) with ≥1 chronic conditions (solid line)	1.00	0.97 (0.73, 1.28)	1.03 (0.78, 1.37)	1.06 (0.78, 1.43)	0.0543
“Healthy obesity” [Obesity (BMI≥30) with no chronic conditions] (dashed line)	1.00	1.18 (0.88, 1.58)	1.64 (1.19, 2.27)	2.45 (1.82, 3.28)	<0.0001
“Unhealthy obesity” [Obesity (BMI≥30) with ≥1 chronic conditions] (dotted line)	1.00	1.33 (0.98, 1.82)	1.55 (1.12, 2.15)	2.44 (1.73, 3.43)	<0.0001
