# Supplementary material for: Using partial least squares to identify a dietary pattern associated with obesity in a nationally-representative sample of Canadian adults: Results from the Canadian Community Health Survey—Nutrition 2015
Source: PLoS One. 2021 Aug 5;16(8):e0255415. doi: 10.1371/journal.pone.0255415 (PMC8341606; doi:10.1371/journal.pone.0255415)
Supplement: S5 Table — Results are across quartiles of the simplified dietary pattern score (SDS). (PDF) [file pone.0255415.s005.pdf]

S5 Table. Odds ratios and 95% confidence intervals for the likelihood of "healthy" or "unhealthy" obesity, with normal weight (BMI<30) as reference. Results are across quartiles of the simplified dietary pattern score (SDS).

	Q1 (Ref; Healthiest)	Q2	Q3	Q4 (Least Healthy)	P-trend
Normal weight (BMI<30) with ≥1 chronic conditions (solid line)	1.00	0.83 (0.61, 1.14)	1.14 (0.84, 1.55)	1.25 (0.92, 1.69)	0.5722
"Healthy obesity" [Obesity (BMI≥30) with no chronic conditions] (dashed line)	1.00	1.09 (0.81, 1.45)	1.76 (1.32, 2.34)	1.95 (1.43, 2.66)	<0.0001
"Unhealthy obesity" [Obesity (BMI≥30) with ≥1 chronic conditions] (dotted line)	1.00	0.99 (072, 1.37)	1.44 (0.97, 2.12)	2.24 (1.59, 3.16)	<0.0001
